# 

**Published:** 1846-06

**Authors:** 


### WESTERN JOURNAL

OF

### MEDICINE AND SURGERY.

EDITORS.

---

DANIEL DRAKE, M.D.,

PROFESSOR OF PATHOLOGY AND THE PRACTICE OF MEDICINE IN THE MEDICAL INSTITUTE OF LOUISVILLE:

LUNSFORD P. YANDELL, M.D.,

PROFESSOR OF CHEMISTRY AND PHARMACY IN THE SAME:

THOMAS W. COLESCOTT, M.D.

### INDEX TO VOL. V.

[NEW SERIES.]

---

A

Abortion, 9, 98.
Acid, hydrocyanic, poisoning by, 415.
Acid, oxalic, poisoning by, 179.
Academy of medicine and French King, 536.
Acid rain, 257.
Ammonia phosphate in gout, 252.
Asylum, lunatic, Georgia, 177.
" " Bloomingdale, 232.
" " Pennsylvania, 235.
" " Kentucky, 269.
Anatomy, pathological, 28, 112, 315.
Acetate of lead, 345.
Aneurism of subclavian, 450.
Animalcules in skin, 357.
Animal chemistry, 401.
Arm and shoulder presentations, 59.
Arsenical injections, danger of, 52.
Artificial teeth, 540.
Asthma, hay, 143.

B

Baker, Dr. Abner's insanity, 11, 90.
Belladonna in hernia, 53.
Beef gall in constipation, 194.
Bichat, honors to, 258.
Bird on urinary deposits, 100.
Bilious colic, enemata in, 259.
Blood, food on, 255.
Boston waters, analysis of, 362.
Boling's case of cataract, 387.
Brain, wound of, 261.
Brocchieri styptic, 258, 271.
Bronchus, foreign body in, 151.
Bronchial affections, warmth in, 358.
Brodie's lectures on surgery, 409.
Buck on fever, 1.
Budd on the liver, 134, 201,
Bunions, 237.

C

Caldwell's case of stricture, 494.
Carpenter's Physiology, 526.
Carroll's case of narcotism, 6.
Castration, 549.
Cataract, 387.
Cataract cured after long blindness, 19J.
Cerebral diseases, 447.
Chapman's Lectures, 523.
Chemistry by Hoblyn, 242.
Chemistry, Turner's, 526.
Children, pneumonia of, 53.
Children, diseases of, 416.
Chilblains, 540.
Chorea, 143.
Churchill's midwifery, 240.
Clairvoyant's suit for fees, 180.
Cough, whooping, 144.
Creation, Vestiges of, 182.
Creosote in dysentery, 52.
Cold water in erysipelas, 444.
Cross's letters from Europe, 185, 277.
Croup, turpeth mineral in, 61.
Corns, 237.
Cornea, opacity of, 450.
Colic, enemata in, 259.
Constipation, beef gall in, 194.
Convulsions, 146, 460.

D

Davy, Sir Humphrey's case, 170.
Dawson on diet, 286, 369, 462.
Diet in health and disease, 286, 369, 462.
Dictionary of practical medicine, 414.
Diseases of children, 416.
Dropsy, sugar in, 151.
Dysentery, creosote in, 52.

E

Electricity in hydrocele, 143.
Electricity to excite uterine action, 171.
Entomology, 530.
Epilepsy, 145.
Ergot of rye, 49.
Ergot in uterine hemorrhage, 92.
Erysipelas, cold water in, 444.
Ether, sulphuric, in enlarged spleen, 147.
Exanthemata and menstruation, 445.

F

Faith in the cure of disease, 170.
Fee's case of hemorrhage, 395.
Fever, 1, 482.
Fever, puerperal, 245.
Fevers, western, 534.
Fever in Africa, 539.
Flourens on phrenology, 225.

Food, its effects on the blood, 255.
Fordyce on Fever, 524.
Fox on the teeth, 527.
Franklin medical college, 452.
French medical congress, 160, 161.
Fry's case of ileus, 458.

G

Georgia lunatic asylum, 177.
Gerhard on the chest, 523.
Graves on pneumonia in children, 53.
Gross's pathological anatomy, 28, 112, 315
Gonorrhœa. 257.
Gout, phosphate ammonia in, 252.
Gordon's obstetrical cases, 390.

H

Harris's case of pneumonia, 198.
Harris on fever, 482.
Harrison's case of hemoptysis, 459.
Hay asthma, 143.
Heart, diseases of, rheumatic, 345.
Hemiplegia, 145.
Hernia, belladonna in, 53.
Hemorrhage, uterine, 92, 145.
Headache, 256.
Heart of a cow, needle in, 149.
Hematemesis, 196.
Hemorrhage from the bowels, 395.
Hereditary predisposition, 169.
Hemoptysis, 459.
Hoblyn's chemistry, 242.
Homœopathy, 506.
Hospital, Pennsylvania, 449.
Hydatids, uterine, 93.
Hydrocele, electricity in, 143.
Hydrothorax, 549.

I

Ileus, 346, 458.
Infantile diseases, 267.
Insanity from fright, 143.
Introductory lectures, 90, 179.
Iodide of iron, 149.
Iodine in porrigo, 460.
Iron, iodide of, 149.
Iron, carbonate, in uterine hemorrhage, 246.
Issues on the scalp in brain diseases, 447.
Johnson, Dr. James, 172.

L

Labor, obscure presentations in, 148.
Latin prescriptions, 271.
Laudanum in delirium, 354.
Lectures, introductory, 90, 179.
Lectures on surgery, 409.
Lightning, effects of, 162.
Lithotripsy, 253.
Liver, diseases of, 134, 201.
Liver, effects of quinine on, 145.
Longings of pregnant women, 256.
Lucifer matches, 542.
Lyle's case of hematemesis, 196.

M

Medicine, forensic, 416.
Medical classes, 273.
Medical congress of France, 160, 161.
Medical convention, N. York, 354, 366, 544, 545.
Medical Institute, commencement in, 361.
Medical obedience, 363.
Medical profession in U. S., 364.
Medicine, hypotheses in, 366.
Mental faculties, suspension of, 153.
Meteorological table, 276.
Midwifery, Churchill's, 240.
Midwifery, Velpeau's, 242.
Milksickness, 184.
Miller's Surgery, 526.
Memphis medical college, 453.
Menorrhagia, 459.
Menstruation and the exanthemata, 445.
Morphia, poisoning by, 6.

N

Narcotism by sulphate morphia, 6.
National medical convention, 354, 356, 457 544, 545.
Needle found in the heart of a cow, 149.
Nelson's case of abortion, 9.
Nerves, Neill on, 91.
Nervous system, 446.
Neuroses, 186.
Neuralgia, facial, tobacco in, 193.
Nitrate silver, 450.

O

Obstetric cases, 352, 390.
Odors, diffusion of. 542.
Ointment for chilblains, 540.
Opium in rheumatism, 543.
Osborne's case of abortion, 98.
Oxalic acid, death from, 172.

P

Pathological anatomy, Gross's, 28, 112, 315.
Phenomena of respiration, 66.
Placenta prævia, 57, 535.
Premature interments, 545.
Porrigo, iodine in, 460.
Phrenology examined, 225.
Phthisis pulmonalis, spontaneous cure, 188.
Placenta extraction before the child, 264.
Pneumonia in children, 53.
Pneumonia typhoides, 198.
Poisoning by oxalic acid, 172.
" by urine, 158.
" by hydrocyanic acid, 415.
" by bitter almonds, 431.
" by morphia, 6, 433.
" by copper, 439, 441.
" by poppy heads, 435.
" by hemlock, 435.
" by arsenic, 436.
" by quinine, 440.
" by mercury, 437.
" by corrosive sublimate, 438.
" by sulphuric acid, 439,
" by savin, 440.
" by zinc. 439.
Polypus of the bowels, 395.
Potato disease, 539.

Pneumonia, 500.
Presentations, arm and shoulder, 59.
Puerperal fever, contagiousness of, 245.
Puberty in the African, 150.
Purpura hemorrhagica, 141, 163, 500.

Q

Quinine in purpura, 141.
Quinine, effects on the liver and spleen, 145.
Quinine, morbid effects of, 174.

R

Report on the progress of surgery, 67.
Report on forensic medicine, 447.
Respiration, phenomena of, 66.
Rheumatism, 345, 539, 543.
Rupture of stomach, 537.

S

Self-emasculation, 549.
Simon's animal chemistry, 401.
Small-pox, epidemic, 183.
Stilwell's case of purpura, 500.
Stewart on diseases of children, 416.
Stricture of the urethra, 494.
Stomach, rupture of, 537.
Sugar in dropy, 151.
Surgery, progress of, 67.
Surgery, by Liston, 243, 244.
Suicide in France, 268.
Syphilis, 347, 448.

T

Tape worm, 51.
Teeth, artificial, 540.
Tobacco, mysteries of, 181.
Tobacco in neuralgia, 193.
Tobacco in ileus, 458.
Turpeth mineral in croup, 61.
Turpentine in purpura, 163.

U

University of Maryland, 453.
University of Louisville, 361.
United States, medical profession in, 454.
Urethra, stricture of, 494.
Urinary deposits, 100.
Urine, poisoning by, 158.
Uterine hemorrhage, ergot in, 92.
" " iron in, 246.
Uterine hemorrhage, 347.
Uterine hydatids, 93.
Uterine action by electricity, 171.

V

Vaccine aphorisms, 272.
Vaccination, 144.
Voyage of a naturalist, 457.

W

Weather of the past winter, 89.
Western Lancet, 85.
Western fevers, 534.
Wound of the brain, 261.

### TO SUBSCRIBERS.

We regret to find that, notwithstanding unusual expenditures upon the present volume of this Journal and its very material improvement, our subscribers have this year been more than usually remiss. We would give notice that all who may not remit promptly shall be forthwith and constantly harrassed with every form of dun until they are worried out. Their names shall appear in a black list on the back of the Journal, and, simultaneously, they shall be called upon and persecuted by duns of every kind, travelling agents, local agents, constables, sheriffs, and catchpoles. We are shamefully deprived of our dues and we mean to have justice done us.

☞Receipt by mail, at our risk, postpaid.

PRENTICE & WEISSINGER.

### PROSPECTUS OF THE SECOND SERIES

OF THE

### RECEIPTS OF MEDICAL JOURNAL FOR MAY, 1846.

| | |
|---|---|
| Dr. J. O. Freemaster, Warrenton, Ala., | $10 00 |
| Dr. B. D. Finy, Sandy Spring, Tenn., | 5 00 |
| Dr. E. D. Wheeler, Memphis, Tenn., | 5 00 |
| Dr. J. L. Bond, Shady Grove, Tenn., | 5 00 |
| Dr. S. Gregory, Stephenson, Ill., | 4 87 |
| Dr. S. McDaniel, Clarksville, Tenn., | 10 00 |
| Dr. A. B. Duval, Hanna's P. O., | 5 00 |
| Dr. D. G. Gregory, La Grange, Texas, | 5 00 |
| Dr. W. Boyle, Oakland, Miss., | 5 00 |
| Dr. P. Wallace, Massillon, O., | 5 00 |
| Dr. A. Kimbal, Ft. Henderson, Ala., | 10 00 |
| Dr. J. B. Berry, Hartford, Ky., | 10 00 |
| Dr. B. D. Hodge, Cartersville, Miss, | 10 00 |
| Dr. R. W. Saffold, Haynesville, Ala., | 5 00 |
| Dr. A. B. Dodd, Bolivar, Miss., | 5 00 |
| Dr. S. Thompson, Albion, Ill., | 5 00 |
| Dr. T. B. Lilly, High Grove, Ky., | 5 00 |
| Dr. L. Shanks, Memphis, Tenn., | 10 00 |
| Dr. J. Travis, Martin's Creek, Ky., | 5 00 |
| Dr. W. C. Sherrill, Carrolton, Ky., | 5 00 |
| Dr, J. Drane, New Castle, Ky., | 5 00 |
| Dr. G. Forest, Lebanon, Ky., | 10 00 |
| Dr. B. Spaulding, Lebanon, Ky., | 5 00 |
| Dr, Sweeny, Dripping Spring, Ky., | 10 00 |
| Dr. Anderson, Flemingsburg, Ky., | 5 00 |
| Dr. J. C. Whitterage, New Paris, O., | 5 00 |
| Dr. D. Cox, New Paris, O., | 3 00 |
| Dr. Richards, Cincinnati, O., | 10 00 |
| Dr. Thrall, Gambier, O., | 20 00 |
| Dr. Falconer, Hamilton, O., | 8 00 |
| Dr. R. Haymond, Brookville, Ind., | 5 00 |
| Dr. F. Cannon, Jackson, Mo., | 10 00 |

